# “Where There Is Light, There Is Also Darkness”: Discussing Young Adults’ Willingness to Disclose Data to Use Wearables and Health Applications—Results from a Focus Group Study

**DOI:** 10.3390/ijerph19031556

**Published:** 2022-01-29

**Authors:** Isabell Koinig, Sandra Diehl

**Affiliations:** Department of Media and Communications, University of Klagenfurt, 9020 Klagenfurt am Wörthersee, Austria; sandra.diehl@aau.at

**Keywords:** privacy, privacy paradox, privacy management, mHealth/eHealth

## Abstract

In recent years, the Internet of Medical Things (IoMT) has gained momentum. This development has only been intensified by the current COVID-19 crisis, which promotes the development of applications that can help stop the virus from spreading by monitoring people’s movements and their social contacts. At the same time, it has become increasingly difficult for individuals to control the use of their private data by commercial companies. While Internet users claim to be highly interested in protecting their privacy, their behaviors indicate otherwise. This phenomenon is discussed in literature as the so-called privacy paradox. The existence of the privacy paradox has also been confirmed by previous studies, which found individuals’ claims and actions to contradict one another. The present study investigates the following research questions: (1) What significance do individuals attribute to protecting their privacy, with a special focus on the health sector? (2) To what extent are they willing to grant commercial parties access to their data in order to use applications in general and health applications in particular? Results from seven focus groups with 40 respondents aged 20–30 years were conducted in an urban setting in Austria in late 2019. The respondents’ inputs are meant to provide answers to these questions. The results indicate that, overall, the young generation is well-informed about the growing data collection and is quite critical of it. As such, their willingness to share information in the health context is only moderately pronounced. Thus, only a moderately pronounced privacy paradox can be detected for the health sector when compared to other sectors. In conclusion, implications and directions for further research are addressed.

## 1. Introduction

Referring to a form of extended Internet connectivity, the Internet of Things (IoT) is immersed into our everyday lives, assisting us in more efficiently managing our daily routines. IoT technology is featured both at work and at home [1,2], for example, in the form of smart offices, smart homes or smart watches. The IoT takes on an elaborate role by embedding technology such as radiofrequency identification (RFID) in smart objects [3], which can communicate with other virtual objects when provided with an appropriate infrastructure [4,5].

The health-care context has not been left unaffected by these developments, where smart health gadgets (e.g., diabetes applications) and fitness applications (e.g., workout applications) constitute parts of the Internet of Medical Things (IoMT). The IoMT does not only lead to a collaboration between disease management and advanced care coordination, but also to more personalized health care and patient empowerment [6]. Other terms used in this context concern mobile health (mHealth), health information technology (HIT), telehealth, telemedicine, or personalized medicine [7]. With more than 40% of all health technology being IoMT-related by 2020 [8], questions regarding privacy are becoming more pressing [9]. Due to the current COVID-19 crisis, the topic has become even more relevant as all over the world software developers are trying to develop applications that can help to stop COVID-19 (e.g., “Stop Corona” in Austria), but also allow for intensive tracking of people’s whereabouts and their personal contacts, causing privacy advocates to protest and revolt.

The Internet has literally turned individual lives into an “electronic panopticon“ [10], where privacy seems hard to be upheld. Against the commercialization of private data, the protection of individual privacy becomes harder to be put into practice. In a broad sense, privacy refers to “the access of one actor (individual, group, or organization) to another” [11]. It has also been described as a “state with limited access to one person” [12] or “to [personal] information” [13]. In a nutshell it alludes to “what people conceal and reveal or what others acquire and ignore” [11]. Notwithstanding the existing fears, a general carelessness regarding the protection of private data can be observed. Amongst the general population, only slightly more than one quarter of individuals protect their privacy by, e.g., changing social media’s privacy settings (27%), not storing any personal or sensitive data (26%) as well as using encryption to protect their data (13%; [14]). Therefore, while individuals claim to be highly concerned with maintaining their privacy [15], their behaviors contradict their claims.

In literature, this phenomenon is frequently referred to as the “privacy paradox” [16,17]. This also applies to younger people, claimed to be a particularly vulnerable population. While some studies have investigated the privacy paradox in general (e.g., [16,17]), there is only scarce research related to the privacy paradox in the health sector. To the authors’ knowledge, empirical studies investigating young people’s attitudes toward data security when using wearables and health applications are missing from the academic discourse. The study at hand tried to reduce this research gap.

The empirical study presented in this paper focused specifically on the area of digital and mobile health, setting out to investigate the degree of importance individuals attribute to protecting their privacy as well as individual privacy protection strategies. Moreover, the extent to which individuals between the ages of 20 and 30 years are willing to grant commercial parties access to their private data to use digital health services and applications was put to the test. To this end, seven focus groups were conducted in Austria to explore individual user experiences and perceptions. In conclusion, results from the 40 participants were thematically grouped and summarized before deriving implications and highlighting directions for future research.

## 2. Theoretical Background

### 2.1. Privacy as a Topic of Relevance and the Privacy Paradox

In recent years, it has become increasingly difficult for individuals to control the use of their private data by commercial companies [17,18]. This aspect was also thematized by previous scientific studies which showed that people are extremely controversial about their privacy. In literature, this phenomenon is discussed as the privacy paradox [13,16,17,19,20,21]. In detail, this term refers to the fact that “users may express concerns and fears about their privacy, but at the same time behave in a manner that appears to contradict their statements” by disclosing personal data [17]. According to a study from 2015, the majority of the respondents (87%) openly admitted not to hesitate to grant commercial providers access to their data in order to use their online services or applications free of charge [22]. In another study, only 3% of all the respondents claimed that they felt indifferent as to what happened to their data [23]. The number of those who feared an inappropriate and unauthorized use of their data was considerably higher (66%; [23]). According to a study on data security, Germans’ fear of other people getting insights into sensitive and personal data (e.g., health data) was the second most possible reason why data were not or only hesitantly released [24]. Data from the same year revealed an ever-increasing privacy paradox: even though 98% of Germans claimed to be committed to protecting their privacy, more than 70% of the respondents indicated to grant social networking services (e.g., WhatsApp) access to their data several times a week [25]. This behavior seems to be indicative of “pragmatic carelessness” in dealing with personal data [26], although data protection is vehemently demanded at the same time [27,28,29]. In recent years, privacy concerns and protection have evolved into an area of tension [30]. The fact “that users may express concerns and fears about their privacy, but at the same time behave in a way that appears to contradict their statements” by disclosing personal information is expressive of a concerning trend [17]. Perceived as a “behavioral paradox of individual information processing” [21], the privacy paradox can be explained by different behavioral heuristics and theories.

Following Tversky and Kahneman [31], individual decision-making is characterized by uncertainty, takes place under time pressure and is influenced by individuals’ inability to deal with large amounts of information. The authors introduce several heuristics of thinking, whereby two forms are of relevance to the study at hand. The *affective heuristic* postulates that individuals arrive at decisions in the spur of the moment and thus have a tendency to underestimate the risks associated with their decisions [32]. This was found to be particularly true if they hold positive attitudes towards the object affected by their decision. However, if they hold negative attitudes, the reverse effect occurs: the risks associated with the decision are overestimated [17,33]. Consequently, if users really want to use a (health) application, they are more likely to underestimate possible risks.

According to the *availability heuristic* [34], the probability of certain decision outcomes is presumed to be higher if the individual is able to relate to them easily. If results in the far future are anticipated, the consequences associated with the decision tend to be weakened because they are hard to estimate (“hyperbolic discount” [20]). Consequently, individuals rate the advantages presented to them today when they use a (health) application as more important than possible disadvantages that might result from the usage and the associated data disclosure at a later point in time [17]. 

Two additional theories might be able to explain why individuals are either willing or unwilling to disclose their personal information in the digital health context. As part of the *utility maximization theory* [35,36], individuals strive to achieve an optimal outcome. Thus, the “added value” that the disclosure of data brings with it outweighs potential risks [37,38]. The resulting added value is called the *privacy calculus*—i.e., the costs of a potential privacy violation are lower as compared to the benefits of using an application or related services [37,39,40]. One theory that takes the particularities of the digital environment into account is the *technology threat avoidance theory* [41,42]. The theory asserts that if individuals are susceptible to the severity of technology threats, they will actively try to avoid this technology to reduce the likelihood of any potential harm being imposed on them.

### 2.2. Privacy Paradox Amongst Young Adults

There are different forms of privacy regulation. *Privacy by design* postulates that privacy—as a value—has to be taken into account in the application development and design process already. *Privacy by default* describes a measure, according to which a commercial party is only granted access to data that can be used for specific (limited) purposes [43]. In the health context, this is in line with the General Data Protection Regulation (GDPR), according to which data can only be used for explicitly stated purposes [44]. *Privacy as forsaken* alludes to the fact that individuals lose control over their data as soon as they post them online [45]. The last aspect has been found to be particularly expressive of the current youth’s mindset, for whom two additional categories have been identified: *privacy as social* and *privacy “in your own hands”* [45]. According to the former, adolescents decide not to post any information that is of concern, while also trusting others not to use the personal information they share against them. The latter—*privacy “in your own hands”*—presupposes individualized responses to privacy [45].

In the digital context, individual behavior surrounding the use of new technology is characterized by evaluating both the benefits and (potential) risks of use [46]. The paradoxical behavior mentioned above is claimed to hold specifically true for teens and young adults. Barnes [47], for instance, discovered that “adults are concerned about the invasion of privacy, while teens freely give up personal information […] because often teens are not aware of the public nature of the Internet”. Adolescents and young adults are renowned to have a habit of “oversharing”, i.e., disclosing a lot of sensitive, personal information and not utilizing any privacy management strategies [46,48]. Previous studies on online usage behavior and privacy management produced conflicting results and have shown that user behavior is quite hard to predict. While some studies found no relationship between users’ age and privacy concerns [49,50,51], other scholars reported that younger people are “more likely to know and use privacy protection strategies than older consumers” [52]. Another set of studies claims the opposite to apply: Paine et al. [53] revealed individuals under the age of 20 to be less concerned about their privacy than individuals over the age of 20. Similar findings were also produced by Dommeyer and Gross [54] and James [45], who found younger people (“tweens”) to be “naïve to the effectiveness of [privacy management] strategies”. 

For this reason, Blank et al. [55] urge for a redefinition of the privacy paradox and introduce the “new privacy paradox”. This “new privacy paradox” alludes to the fact that social networking sites have become so embedded in users’ social lives that they must disclose information on these sites in order to use them. Nonetheless, these sites do not provide adequate privacy control measures, which challenges users. The same applies to applications. Although recent years have seen some significant improvement in terms of (advanced) privacy settings [48], progress is still slow. Interestingly, when users are given more control over their privacy, only a “vanishingly small number of users change the […] default privacy preferences” [56]. In spite of low trust in social media to protect their private data [57], only 40% of US users reportedly enforce stricter privacy regulations on social media [58]. This might be conditioned by the adolescents’ “nothing to hide” attitude, according to which privacy is neither a sensitive topic nor an important value to protect [47,48]. Some authors have even go as far as to claim that the youth have become more shameless and do not worry about their privacy at all [59,60,61]. This suggests that the privacy paradox is less pronounced in the daily use of social media, but has not yet been explored in the health sector.

### 2.3. Privacy Paradox in the Health Sector

In the digital health context, the collection and use of digital health data has been increasingly thematized [62,63,64,65,66]. The increasing “universal datafication” [67] has resulted in the continuous invasion and/or loss of privacy, which contradicts the high value of privacy attributed to health information in Western societies [68]. Lupton and Michael [69] refer to this practice as “digital dataveillance”, which leaves individuals in the dark as to which data are recorded and for what purposes [70]. 

Individuals have started to excessively use gadgets and applications that support the so-called lifelogging trend [71,72,73], which describes the continuous recording of one’s daily activities by means of digital devices or computer applications. While lifelogging grants individuals a sense of empowerment [74], it presents a form of self-surveillance during which a large amount of data is collected. Application users are turned into “data subjects” who are permanently surveilled and monitored [75]. Medical data are—all of a sudden—is of relevance to commercial parties, e.g., insurance companies that have started to base their rates on the individuals’ health status and medical records [18]. In the process, a shift of power occurs, and responsibility for managing health data and related privacy settings lies with the individual—the “responsible and healthy subject” [76] (see also James’ [45] privacy “in your own hands”). 

While digital technology is on the rise, legal requirements have been found to lag behind [67]. Due to their novel and innovative product character, numerous medical gadgets (e.g., wellness applications) cannot be categorized, challenging current legal regulations [44]. As technical developments are hard to predict, innovative health technologies were purposely not addressed by legal documents in order to avoid the continuous adaption of laws [67]. Even though harmonization was meant to be achieved, authorities are continuously reviewing cases, especially when it comes to the processing of sensitive health data. This is conditioned by the fact that health care is either subject to the individual states’ jurisdiction [44,67] or, in case of selected service offerings, even exceeds national borders [44,77]. 

Health data are often collected intentionally (e.g., through self-tracking devices or applications), but in other instances, health data are recorded unintentionally (e.g., when using the smartphone for tracking purposes [78,79]). This is critical given that health issues have been found to be a highly sensitive and private matter. As a consequence, individuals value the privacy, anonymity, and confidentiality of their health-related data [80]. As health data qualify as “sensitive data” (Art. 9, GDPR), their use is limited (“purpose limitation” (Art. 5 1c, GDPR)). This implies that any health-related data or information “can only be processed for specific, explicit, and legitimate purposes” [44]. Nonetheless, it appears as if the protection of health data requires some elaborate considerations and active efforts on behalf of individuals. The study presented in this paper intended to investigate these important aspects through a qualitative focus group study. 

## 3. Empirical Study

### 3.1. Study Purpose 

The present empirical study focused specifically on privacy and data protection in the context of digital and mobile health, which has received only limited attention to date [64,65,66]. One study explicitly addressing privacy in the context of smartwatch use talked about the so-called “smart wearables-privacy paradox” [46]. In their article, the authors built on the previous research that had dealt with the “personalization-privacy paradox” [36,81,82,83] and found users to separately evaluate the benefits and risks associated with smartwatch use [36]. Their findings support previous research [82], according to which the perceived benefits explain both users’ continued use and adoption of smartwatches [83,84]. 

Moreover, adolescents’ and young adults’ privacy management strategies have hardly been put at the center of investigations, let alone in a digital setting [85] or in the health context. Hence, the present study sought to answer the following research questions:

1. How important is the protection of individual privacy (a) in general and (b) in the health-related context in particular?
2. Which privacy protection strategies do individuals employ in the digital (health) context?
3. To what extent are individuals willing to disclose personal data in order to (a) use specific services or applications in general and (b) health-related services (e.g., wearables, mHealth applications, or the electronic health record) in particular?

Using these research questions, we set out to scrutinize the extent to which the privacy paradox can be identified for the digital health context.

### 3.2. Study Design and Population 

To answer these research questions, seven focus groups with individuals aged 20–30 years were conducted in an urban setting in Austria. As research on the topic is scarce, we decided to take a qualitative approach to investigate the motives, practices, concerns, and strategies of the participants in depth. The focus was put on this age segment, that has grown up in a digitally immersive environment (“digital natives” [86]). Moreover, it is particularly the young generation who are not only interested in health and fitness [87], but also already use health-supporting applications or gadgets [88,89]. In Switzerland, this segment has been identified to hold the largest market potential [90]. Moreover, this age segment has been found to hold little reservations when it comes to sharing data or personal information (73% [91]). Similar results were obtained for the US [92]. 

In total, seven focus groups with 5–7 people aged 20–30 years each were conducted. The average age was 23.4 years. The total sample comprised responses by 40 respondents, 20 of which were female and 20 of which were male. Approximately one third of the participants were students. The subjects were recruited in August and September 2019 by two trained researchers via email and were offered an incentive for their participation. The researchers did not only set up and moderate the focus groups, but also fully transcribed the audio files. In general, interviews lasted between 40 and 75 min. 

Data analysis was conducted using QCAmaps, an online software tool that is free of charge. The qualitative data analysis was based on reflexive thematic analysis [93], which allowed us to identify patterns of meaning in the dataset that were relevant to the research questions [94]. For the study at hand, we were particularly interested in respondents’ attitudes towards new health technologies, the importance they attribute to privacy in general and in the health context, as well as their willingness to disclose personal information to use selected health gadgets or applications.

In terms of analysis, an inductive approach was used, meaning that in the process of data analysis, we came up with thematic categories, which were based on respondents’ statements [95]. Themes then referred to analytic output derived in the process of coding. Afterwards, these themes were refined and grouped into the main themes and the subthemes [96]. 

The procedure followed the steps proposed by Clarke et al. [97]. In the first step, we familiarized ourselves with the data, then continued with the coding of the data. In the next step, the first themes were identified. The themes were reviewed before the final main themes and subthemes were defined and named. Finally, we wrote up important quotes in the respondents’ own words to support the main themes and subthemes (in italics). 

We identified five main themes, that are detailed in the Results: (1) importance of privacy and general privacy management, (2) privacy protection strategies, (3) importance of health and health data, (4) digitization of health: evaluations, and (5) the privacy paradox in the digital health context. An overview of the main themes and the subthemes, together with the corresponding quotes, can be found in Table A1 in Appendix A. The interview guideline used for the focus groups can be found in Table A2 in Appendix A. 

## 4. Results

In line with findings from previous research, the young adults recruited for the present study stressed the relevance of new technology to their daily lives. On the European Commission’s Digital Economy and Society Index, Austria ranks 12th with regard to the country’s digitalization level [98]. While the country’s scores in connectivity and digital public services are above average, progress in the areas of the integration of digital technology and Internet use are subpar [98]. 

Having grown up in a digitally immersive environment, the respondents unanimously stated spending a large amount of their day with new technology. Being part of the generation of digital natives, they were also familiar with a number of digital offerings, both related and unrelated to the health-care context. 

In the following, the previously identified main themes and subthemes are introduced and supported by the respondents’ quotes and statements. For this purpose, the statements (originally given in German) were translated into English.

**Theme** **1.** *Importance of privacy and general privacy management.*

Subtheme 1-1: Responsible participation and anonymity

When inquired as to how they handled their private data, the largest proportion of the respondents indicated that privacy was very important to them. The respondents stated to be *very careful* (male, 21 y/o) and *consciously handle personal information* (female, 23 y/o). One respondent even referred to it as *responsible handling* (male, 25 y/o). 

The participants also agreed that in order to ensure that not all information on them would be publicly available, they had to *take responsibility for [themselves]* (female, 23 y/o). For instance, this involved *adjusting privacy settings to regulate what is online and who can read it* (female, 21 y/o). For the participants inquired, privacy entailed *not disclosing any personal information to unknown or third parties* (female, 22 y/o) as well as *staying anonymous* (male, 22 y/o). One respondent even shared:

*It is important to me that data and images to which I hold the copyrights cannot be used without my permission.*

(female, 20 y/o)

Given the fact that young adults consider privacy an important value that needs to be upheld, they utilized a number of protective strategies to ensure the confidentiality of their information. There is agreement that protecting their privacy is *quite problematic*, as *more and more data are required* (female, 23 y/o).

**Theme** **2.** *Privacy protection strategies*.

In the context of privacy protection, three subthemes emerged:

Subtheme 2-1: Personal agency

Since the protection of their private information was paramount to individuals, they indicated doing so proactively. Example actions as provided by the respondents included, but were not limited to, *deleting cookies* (female, 25 y/o; male, 21 y/o; female, 23 y/o), *deactivating GPS and tracking services* (female, 20 y/o), *searching in the private mode* (female, 20 y/o), *providing as little information as possible* or even *false information* (female, 26 y/o; female, 23 y/o). Besides *studying privacy settings in detail* (female, 21 y/o), the participants also emphasized the necessity to *use different passwords that are not easy to crack* (female, 23 y/o). The *passwords*, of course, *need to be changed regularly* (male, 23 y/o) and *not saved automatically* (male, 20 y/o).

Subtheme 2-2: Software-related solutions

To a limited extent, software-related solutions were seen as aiding individuals in protecting their privacy. One respondent noted:

*I think it is important to use a good antivirus program.*

(female, 26 y/o)

Subtheme 2-3: Limited use of applications and devices

Additional privacy protection measures as introduced by the respondents had to do with either *limiting the downloading of applications* (male, 23 y/o), *deleting the browsing history* (female, 23 y/o), or *avoiding sites that are not trustworthy* (female, 23 y/o). More than half of the respondents also stated *reading data protection declarations carefully* (female, 23 y/o) or reducing their online visibility on social media by *setting profiles to private* (female, 22 y/o). One focus group participant also claimed:

*I attentively examine any contact inquiries or emails I receive.*

(female, 23 y/o)

**Theme** **3.** *Importance of health and health data.*

Before inquiring about the respondents’ privacy management strategies in regard to digital health services including wearables and health applications, it was important to establish the relevance respondents attribute to health. In general, the respondents agreed that health is something very important, stressing that *it is the only thing that somehow cannot be fixed with money, so to say* (female, 26 y/o). One respondent remarked that *while everyone defines health differently, it is something that is encountered on a daily basis: when you see fitness models or something like that, they somehow set the trend so that you look after your body, eat a healthy diet,* etc. (female, 26 y/o). Specifically, the availability of fitness and health-related applications and the health data they provide render health a very important topic (male, 21 y/o).

**Theme** **4.** *Digitization of Health: Evaluations.*

As discussed in the previous section, health is seen as a valuable asset. Digital developments as brought about by the introduction of the IoMT have also proven to impact the health sector. The respondents perceived these developments critically. In consequence, two subthemes were identified:

Subtheme 4-1: Advantages of digital health

The respondents indicated perceiving the digitalization of the health-care sector and health-care services as favorable and stressed a number of points in favor of these developments, such as a *faster and time-independent transfer of data* (female, 20 y/o; male, 24 y/o), *reduced health-care costs and cheaper care* (female, 20 y/o), as well as the *possibility to access health information at a glimpse*, which is brought about by *newly enabled transparency* (female, 25 y/o; female, 21 y/o). This *collected medical history also makes it easier for doctors to diagnose their patients* (female, 26 y/o; female, 23 y/o) and has proven to be particularly *beneficial in cases of a medical emergency* (female, 22 y/o), where *mistreatment can be prevented since medical records are available in digital form* (female, 23 y/o). The potential of *personalized recommendations* (male, 21 y/o) was highlighted as well. Some respondents even judged the introduction of digital health information in terms of their economic advantage, proposing that *analog storage is no longer necessary* (male, 23 y/o) and that because of digital health records, the *loss of data can be prevented* (female, 23 y/o). Digital health services were further found to allow for *reminder functions* (female, 23 y/o), which—together with some more elaborate health applications—would lead to *a more “conscious” experience of health* (female, 25 y/o). Nonetheless, health data hold a rather special status; according to one respondent, *health data are what I give to a doctor, not anyone else* (male, 21 y/o).

Subtheme 4-2: Disadvantages of digital health

While a number of advantages was identified in the context of digital health information and services, the respondents did not fail to also point out the downsides of the continuous digitalization of health and health care. First and foremost, they criticized the *increasing dependence on technology* (female, 23 y/o), which would not only come with *high conversion costs* (male, 21 y/o), but also *increase the risk of sensitive data being stolen*, as with an increasing reliance on technology, *the likelihood of viruses increases, too* (female, 23 y/o). 

One respondent also feared that the availability of new data in abundance could benefit companies, while putting individuals at a disadvantage.

*I am afraid that companies could make money off my data. They could resell these data to commercial parties. I do not want that.*

(female, 23 y/o)

Even though not explicitly stated by other respondents, this unease seems to be related to a general uncertainty as to how the data are used and for which purposes. The following statements exemplify this insecurity:

*I do not know what happens to my data.*

(female, 25 y/o)

*I think if we disclose too much, we are vulnerable and prone to abuse.*

(female, 20 y/o; male, 21 y/o)

Overall, the respondents felt that having data widely available may be beneficial to some parties (e.g., physicians, hospitals), while they were opposed to other parties having access to their data, such as their *employers or insurance companies* (male, 23 y/o; female, 21 y/o). One female respondent voiced her concern:

*I feel like if my health data got into the wrong hands, it could cause enormous damage.*

(female, 26 y/o)

The statements given above indicate that while the digitization of health data is seen as beneficial, a lot of reservations exist, rendering digitization in the health context a double-edged sword.

**Theme** **5.** *Privacy paradox in the digital health context.*

As postulated in the beginning of this paper, individuals—and young adults in particular—tend to openly grant parties access to their data if it turns out to benefit them, i.e., the privacy calculus is perceived as sufficiently high and the benefits received outweigh the potential risks. This behavior has become commonly known as the privacy paradox. The present study was interested in uncovering whether individuals would also exhibit such behavior when it came to disclosing their health information to health application or digital health service providers.

For the digital health context, five subthemes could be identified.

Subtheme 5-1: Health data as private data

Surprisingly, while the respondents agreed to freely share their data with commercial parties if the perceived privacy calculus was appealing (e.g., a promo code or a gift card was received in exchange for their data), they were less willing to grant parties access to their health data. One respondent remarked:

*I think psychological and physical well-being is definitely something very private for everyone and it must be protected. I do not think that it has to become public.*

(male, 25 y/o)

Another focus group participant emphasized the sensitive character of health information, stating:

*I believe that such data, if they are so important—like bank data—health data should be better protected.*

(female, 23 y/o)

When inquired about the likelihood of disclosing his personal health records to third parties, another male respondent remarked:

*I would not do it […]. For me, that is something very intimate, so to speak, which is always a little more sensitive, where I need to take extra care.*

(male, 25 y/o)

One user even proclaimed to be always skeptical, accusing commercial parties of doing things not out of benevolence:

*They want to know everything about us. Our data are the most precious information we have. Health data, in particular, are not yet easily accessible. That makes them all the more valuable.*

(female, 26 y/o)

Given the *precious nature of health data* (male, 23 y/o), which has led to the development of the “quantified self” [99], and in which health equals high performance [65], the respondents claimed to hesitate about giving out their health data for fear of discrimination, e.g., from their employers. One respondent noted:

*You might not be hired if they find someone who is healthier than you.*

(female, 21 y/o)

Subtheme 5-2: Granting access for health purposes

The respondents further indicated being unaware and, respectively, unsure as to what parties have access to their health data. Generally, they did not object to granting health-care providers insights into their health data, as expressed by one respondent:

*In principle, I would have no problem with sharing my health data, if only with certain parties and if it results in positive outcomes. So… in the hospital or with my family doctor; they can work well together and help me if they have access to my data.*

(male, 21 y/o)

Disclosing data to parties outside the health-care context, however, did not resonate well with respondents. The following statements support this argument:

*For instance, if you say that my employer also has access to it, I would not be happy because these are personal data and uh... I can well imagine that this is a future scenario, but I would not be pleased at all.*

(male, 21 y/o)

*Personally, I think I am just more careful when it comes to my health data.*

(male, 25 y/o)

With regard to health data, the respondents differentiated between the groups who would get access to their data. While it was acceptable for them to share their data with doctors and other medical personnel, they were reluctant to share information with other groups, especially insurance companies and their employers. This further suggests that the respondents take more precautionary steps to protect health-related data because of their sensitive nature.

Subtheme 5-3: Unawareness as to the value of health data

Previous research has attested to young adults’ willingness to disclose some of their personal data in order to use specific health-related services or applications and, in doing so, contradicting their initially uttered beliefs regarding the relevance of privacy (i.e., the privacy paradox). When inquired about her readiness to disclose personal information, one female student admitted to freely do so:

*Yes, I am a prime example. I always have my watch on. I mean, I do not think that much about it. I always wonder, what would someone find out about me when looking at my data… You really only see my pulse rates and things like that. I do not think they can read much into it.*

(female, 26 y/o)

Two other respondents also claimed to be unaware as to what could be read into their health data or simply preferred the reward they received in return:

*For instance, if they want my height, they can have it. Because then I get something in return that I can use. Like a tailored offer.*

(female, 21 y/o)

*If I go for a run and I have this application, it records everything. The distance I ran, my pulse… I think that is cool. But I do not think anyone can read anything into my data.*

(male, 27 y/o)

Subtheme 5-4: Non-participation as a choice

Another respondent indicated to rather “miss out” on using selected health-related services and applications if it meant releasing too much and too sensitive data. He commented:

*I think if I wanted to use some health service, I would honestly be too lazy to read through the [privacy declaration] pages right away, to whom [the information] is being passed on, or who might have it. I think I would rather say that I would not use it at all, right from the start.*

(male, 21 y/o)

Another respondent’s answer was also linked to subtheme 5-4.

*I would not do it [i.e., disclose data]. For me, that is something very intimate, so to speak, which is always a little more sensitive.*

(male, 25 y/o)

Subtheme 5-5: Usage comes at a price

This willingness to accept sacrifices in privacy was seen to be linked to individuals’ usage motivations, with one respondent noting:

*I think it is always a question of whether you want something or not. If I want to have this health application now, I just get it. And then I agree to the terms of use.*

(male, 25 y/o)

Especially if the perceived risks are low, the respondents were found to be more likely to download applications. One focus group participant shared:

*I cannot name one thing, something like this has never happened to me… that I was somehow excluded or disadvantaged on the basis of my health data. I think I would only get upset if something like that happens to me that is bad. But at the moment I do not really even think about it.*

(female, 20 y/o)

While other respondents based their decisions on previous bad experiences, this participant was unaware of any past happenings and claimed she would re-evaluate the situation should she ever encounter any discrimination based on her recorded health data (female, 21 y/o). A more reasoned answer was provided by another female participant, who observed:

*Somehow, I think that everything has its price. And you just cannot avoid it. If an application or service is appealing, you want it because everyone has it, and if you do not have it, you are excluded. And you simply give in….*

(female, 26 y/o)

In the last example, the added value is stressed, which might lead individuals to compromise on their privacy. 

Overall, the results can be summarized as follows: while the perceived added value (privacy calculus) was sufficiently high for some respondents to compromise on their reservations and disclose some personal information in order to use health-related applications and services, others indicated refraining from using technological health innovations altogether. Thus, the privacy paradox could only partially be confirmed in the health-care context.

Related to the dynamics within the different focus groups, the topic of the study was met with great interest among the respondents and provoked lively discussions. There was agreement on the importance of privacy protection in the health-care context. The views on the topic, however, differed widely: some respondents were very restrictive with their data, while for other participants, the advantages of using health applications or digital health services prevailed. If there was disagreement, divergent viewpoints were discussed by the respondents, and less skeptical respondents could be convinced in some instances, but not always.

## 5. Discussion of the Results and Implications

The study at hand was based on the assumption that insights into individuals’ privacy concerns are crucial to determining the present and future of wearables and mHealth usage. For this reason, a qualitative approach was chosen. Through focus groups, individuals’ attitudes towards, experiences with, and perceptions of wearables and digital health technologies were inquired. 

Following previous research, we scrutinized whether high privacy concerns led users to refrain from using wearables, or whether the perceived benefits of individualized data and personal offerings would increase individuals’ intentions to employ smart gadgets and digital health applications. 

The results of the study show that the young generation is well-informed about the growing data collection in the context of big data [63] and is quite critical of it. When asked about the importance of privacy, the focus group participants freely admitted to employing a wide range of privacy management strategies, such as deleting undesired content, using pseudonyms, offering incorrect information regarding age or location, as well as utilizing advanced privacy settings. These and other strategies confirm results from previous studies from other sectors (for example, [45,59,100,101,102]) and recent opinion polls [103]. 

Concerns about data usage by third parties are limited since many respondents can understand the motives behind this data collection. Through their answers, some respondents indicated making use of commercial parties’ offers (e.g., free Amazon gift cards or promo codes), but were less willing to provide commercial parties access to their health data. Their willingness to disclose data was limited to health-care providers. The respondents were further aware that while they could not prevent all data from being collected due to some preinstalled health applications on their smartphones, they were not willing to pass on the reins completely. Instead, they believe in self-determination and personal agency (*privacy “in your own hands”* [45,74]). This can be explained by the fact that health data are “sensitive data” [44].

In an attempt to derive potential explanations for a lower willingness to disclose health-related information amongst respondents, the *affective heuristic* proved to be useful. If incentives (e.g., promo codes) were awarded, positive effects prevailed in the eyes of individuals, increasing their likelihood of sharing data. In the health-related context, however, negative consequences of data disclosure came to the individuals’ minds first (e.g., data misuse, data sharing with employers or insurance companies); consequently, the respondents held a negative attitude towards disclosing data, and were more reluctant to grant these parties access to their data. This tendency might be explained by the fact that the respondents tended to overestimate the risks associated with data disclosure. The last aspect can also be linked to the *availability heuristic*. The respondents could imagine health data being used for their disadvantage, e.g., by their (prospective) employers or insurance companies. Only if the respondents were unaware as to what commercial parties could read into their health data, their willingness to disclose information was more pronounced. In this case, the participants’ answers were indicative of a *hyperbolic discount*, which postulates that if future consequences cannot be estimated, they tend to be downplayed. In instances like these, the existence of the privacy paradox in the health context could be confirmed. 

Turning to the *utility maximization theory*, individuals will perform a certain action if it is linked to a favorable outcome. However, if they are unable to perceive the added value—in case of the present study, the privacy calculus—they are reluctant to perform such behavior. The majority of the respondents of our focus groups indicated that they failed to see the benefit they would receive from granting health applications—and, thus, commercial parties—access to their data and therefore decided not to use the applications altogether. This indicates that transparency as to what is happening with their data is not a given. Hence, individuals need to be made aware as to how they can restrict the access options to their health data (see also [17]), which elevates the degree of trust they put in applications. This might be perceived as a form of personal empowerment [74] and could reduce the respondents’ concerns at the same time. Likewise, if individuals believe they are in control, their fear of utilizing new and innovative technology for health-related purposes might be reduced (as postulated by the *technology threat avoidance theory*). Moreover, since transparency as to what is happening with individual data (which is regulated by complex yet incomplete legal regulations [44]) is lacking, consumer trust in data processing by commercial parties needs to be strengthened.

Summarizing, based on the findings from the seven focus groups with 40 participants between 20 and 30 years of age, the existence of a privacy paradox for the health sector could be empirically confirmed—at least to a certain degree. The heuristics and theories outlined in the theoretical part proved to be useful in explaining the behavior of the young Austrian adults. Conditioned by the ever-increasing relevance and use of the Big Data [104] and the IoMT [6], privacy and data protection continue to be crucial matters that need to be addressed [9]. Building on our contribution’s title, “Where there is light, there is also darkness”, the benefits of smart technology (light) are often outweighed by the potential downsides (dark), whose gravity might keep individuals from adopting IoMT solutions altogether. While our respondents were clearly interested in digital health offerings—conditioned by their wide availability and ease of use—reservations as to sharing health data with parties other than the intended health-care and health service providers dominated the discussion. Our study results clearly indicate that data privacy in the health sector follows different rules than in other sectors, where data are more willingly shared. Therefore, especially in the health-care sector, transparency about what happens to individual data and for what purpose they are processed must be increased [22,44]. This, for instance, could be achieved through consumer education [105] and governmental involvement, which has limited both the amount of data to be collected (data minimization [44]) and the time of data storage. Until improvement of legal regulations is achieved, privacy remains in the hands of the individual (*privacy “in your own hands”* [45]). 

## 6. Limitations and Directions for Future Research

Despite the merits of our study, there are some limitations that indicate avenues for future researchers. The study at hand was purely qualitative in nature and allowed us to identify some core aspects that could be taken up in a follow-up (quantitative) study. Likewise, while the current project looked at responses from young adolescents (“digital natives”), it might also be worthwhile to scrutinize how the older generation (“digital immigrants”) perceives new technology and whether they hold similar reservations about disclosing their health-related data. Since older people tend to face not only more health problems, but also more serious health problems, we assume that they might have even more reservations about privacy violations than the “digital natives” do. Younger people often have a better health status than older people and might therefore perceive having better control of their health. Future research might want to analyze the perceptions of people with different health status in more detail. Previous research has also highlighted gender to play an essential role in wearables use and uptake (e.g., [106,107,108]); for this reason, it might be worthwhile to discuss results from the gender perspective. In our study, we focused on Austria. To compare cultural particularities and analyze the generalizability of our results, additional countries should be added to the study corpus in the future. Comparable results can be expected from Germany and Switzerland, which are similar to Austria in terms of digitalization. However, it would be interesting to extend the study to the other continents, too. Future studies might also want to consider whether the study is conducted in a rural or urban area, which might affect its overall results and generalizability.

## Data Availability

The data presented in this study are available on request from the corresponding author.

## Appendix A

**Table A1:** Overview of the themes and subthemes (multiple answers were possible; answers are listed in descending order of frequency, with the top answers listed at the top of the respective category).

**Importance of Privacy**	
** Responsible participation and anonymity** **(100% of the respondents) **	- *very careful* and *consciously handling of* personal information (female, 23 y/o; male, 21 y/o) - *responsible handling **of private data* (male, 25 y/o) - *entails not disclosing any personal information to unknown or third parties* (female, 22 y/o) - *protecting my anonymity* (male, 22 y/o) - *problematic, as more and more data are required* (female, 23 y/o)
** Privacy Protection Strategies **	
** Personal agency** **(80% of the respondents) **	- *take responsibility for myself and my data (female, 23 y/o)* - *adjust privacy settings to regulate what is online and who can read it (female, 21 y/o)* - *delete cookies (female, 25 y/o; male, 21 y/o; female, 23 y/o)* - *deactivate GPS and tracking services (female, 20 y/o)* - *search in the private mode (female, 20 y/o)* - *provide as little information as possible or even provide false information (female, 26 y/o; female, 23 y/o)* - *study privacy settings in detail (female, 21 y/o)* - *use different passwords that are not easy to crack (female, 23 y/o).* - *change passwords regularly (male, 23 y/o)* - *do not save passwords automatically (male, 20 y/o)* - *read the data protection declaration carefully (female, 23 y/o)* - *attentively examine any contact inquiries or emails I receive (female, 23 y/o)*
** Limited use** **(15% of the respondents) **	- *limit the downloading of applications (male, 23 y/o)* - *deleting their browsing history (female, 23 y/o)* - *avoid sites I do not trust (female, 23 y/o)* - *reduce my online visibility on social media by setting my profile to private (female, 22 y/o)*
** Software-related** **(5% of the respondents) **	- *use a good antivirus program (female, 26 y/o)*
** Importance of Health **	
**Definition of health** **(100% of the respondents) **	- *everyone defines health differently (female, 26 y/o)* - *it is something that is encountered on a daily basis: When you see fitness models or something like that, they somehow set the trend so that you look after your body, eat a healthy diet, *etc.* (female, 26 y/o)* - *If you are healthy, you are also mentally healthy (female, 23 y/o)* - *For me, being healthy means living with no restrictions (male, 21 y/o)* - *Health is necessary to make for a good quality of life (female, 24 y/o)*
** Health as a “sensitive” topic** **(100% of the respondents) **	- *I think psychological and physical well-being is definitely something very private for everyone and it must be protected. I do not think that it has to become public (male, 25 y/o)*
** Health as a “value”** **(80% of the respondents) **	- *a very important topic (male, 21 y/o)* - *it means to have full control of yourself (male, 21 y/o)* - *it is the only thing that somehow cannot be fixed with money, so to say (female, 26 y/o)* - *health is something very personal (male, 22 y/o)* - *if you feel healthy and are not burdened by sickness (male, 22 y/o)*
** Digital Health **	
** Advantages** **(50% of the respondents) **	- *faster and time-independent transfer of data (female, 20 y/o; male, 24 y/o)* - *reduced health-care costs and cheaper care (female, 20 y/o)* - *the possibility of accessing my health information at a glimpse (female, 21 y/o)* - *transparency (female, 25 y/o)* - *collected medical history makes it easier for doctors to diagnose their patients (female, 26 y/o; female, 23 y/o)* - *beneficial in cases of a medical emergency (female, 22 y/o)* - *mistreatment can be prevented when doctors can turn to digital health information (female, 23 y/o)* - *possibility of getting personalized recommendations (male, 21 y/o)* - *analog storage is no longer necessary (male, 23 y/o)* - *because of digital health records, the loss of data could be prevented (female, 23 y/o)* - *reminder functions (female, 23 y/o)* - *a more “conscious” experience of health (female, 25 y/o)*
** Disadvantages** **(50% of the respondents) **	- *increasing dependence on technology (female, 23 y/o)* - *sensitive health data can be stolen (female, 23 y/o)* - *the danger of viruses increases (female, 23 y/o)* - *high conversion costs (male, 21 y/o)*
** Privacy paradox in the Digital Health Context **	
** Health data as private data** **(90% of the respondents) **	- *For me, it would be important to know who gets access to my health data (male, 21 y/o)* - *health data is what I give to a doctor, not anyone else (male, 21 y/o)* - *I believe that such data, if they are so important—like bank data—health data should be better protected (female, 23 y/o)* - *Health data are precious data (male, 23 y/o)* - *Personally, I think I am just more careful about sharing health data (male, 25 y/o)* - *I do not think I would qualify what an application records as health data. Recording physical data is not health data, in my view (male, 24 y/o)*
** Granting access for health purposes** **(50% of the respondents) **	- *In principle, I would have no problem with sharing my health data, if only with certain parties and if it results in positive outcomes. So… in the hospital or with my family doctor; they can work well together and help me if they have access to my data (male, 21 y/o)* - *I work as a paramedic, and the availability of digital health data has helped us a lot… everyone, we, the hospital... have access to all the data that we need to treat the patient (male, 21 y/o)* - *I think it is positive if doctors pass on health data to other doctors. Or the pharmacist. I would like that (male, 28 y/o)*
** Non-participation as a choice** **(75% of the respondents) **	- *I do not want selected parties to have access to my data, such as my employer or insurance companies (male, 23 y/o; female, 21 y/o)* - *I feel like if my health data got into the wrong hands, it could cause enormous damage (female, 26 y/o)* - *For instance, you might not be hired if they find someone who is healthier than you (female, 21 y/o)* - *I would not do it [disclose data]. For me, that is something very intimate, so to speak, which is always a little more sensitive where I need to take extra care (male, 25 y/o)* - *For instance, if you say, that my employer also has access to it, I would not be happy because these are personal data and uh... I can well imagine that this is a future scenario, but I would not be pleased at all (male, 21 y/o)* - *I am always skeptical. Companies do not to do things out of benevolence. They want to know everything about us. Our data are the most precious information we have. Health data, in particular, are not yet easily accessible. That makes them all the more valuable (female, 26 y/o)* - *I think if I wanted to use some health service, I would honestly be too lazy to read through the [privacy declaration] pages right away, to whom [the information] is being passed on, or who might have it. I think I would rather say that I would not use it at all, right from the start (male, 21 y/o)*
** (Application) usage comes at a price** **(25% of the respondents) **	- *companies might resell data to commercial parties (female, 23 y/o)* - *companies can make money off my data (female, 23 y/o)* - *If you are using a fitness tracker, you are more or less forced to disclose some of your data. You do not have a choice (female, 26 y/o)* - *I put a lot of thought into it. But then again, if you want it, you just agree to it (female, 26 y/o)* - *If you want to use an application, you basically have to disclose everything (male, 27 y/o)* - *I know that data are often recorded unintentionally. For example, I sometimes check my phone’s health application… just to check how many steps I have walked today (female, 22 y/o)*
** Unawareness** **(25% of the respondents) **	- *Yes, I am a prime example. I always have my watch on. I mean, I do not think that much about it. I always wonder, what would someone find out about me when looking at my data… You really only see my pulse rates and things like that. I do not think they can read much into it (female, 26 y/o)* - *For instance, if they want my height, they can have it. Because then I get something in return that I can use. Like a tailored offer (female, 21 y/o)* - *If I go for a run and I have this application—it records everything. The distance I ran, my pulse… I think that is cool (male, 27 y/o)*

**Table A2:** Question guide for the focus groups (translated from German).

Question Guide
***General***
1. Are you concerned about privacy?
2. How important is your own privacy on the Internet to you?
3. Do you take measures to protect your privacy on the Internet? If yes, what measures?
4. To what extent do you disclose personal information in order to use certain services?
How often do you disclose personal details about yourself?
5. What conditions have to be met for you to disclose/share your data online?
6. Do you worry about who is using your data and for what purpose?
***Health and digital health***
7. Health as a topic area is becoming more and more socially relevant. How do you define health?
8. How important is health to you?
9. Digital Roadmap Austria—the digitization strategy of the Austrian federal government—thematizes the expansion of digital offers in the health sector (e.g., a digital vaccination card, a digital health record). How do you feel about these developments?
What are the advantages and disadvantages associated with digital health offerings?
***Digital Privacy Paradox***
10. Imagine you want to use an application in the health sector and you are asked to disclose personal information. How do you react?
11. Due to the increasing social importance of health, more and more health applications and wearables or fitness trackers are becoming available. Do you already use such applications or do you intend to use them in the near future (in the next six months)?
If yes, which?
12. How do you feel about the permanent documentation of health data through wearables and applications?
13. Is it important for you to know who receives and uses your health data?
14. People are often skeptical about disclosing data on the Internet, but they still do so when they receive benefits (price reductions, services, gifts, etc.). Do you recall any instances where this has happened to you?
15. Can you remember one particular instance/situation when you decided to disclose information even though you did not want to?
